# Avoidability of drug-induced liver injury (DILI) in an elderly hospital cohort with cases assessed for causality by the updated RUCAM score

**DOI:** 10.1186/s12877-020-01732-3

**Published:** 2020-09-14

**Authors:** Mohammed Ibn-Mas’ud Danjuma, Hussam Almasri, Shaikha Alshokri, Fadi Khazahia Khir, Ashraf Elmalik, Naim Ghazi Battikh, Ibtihal Mahmoud Hassan Abdallah, Mohamed Elshafei, Haajra Fatima, Mouhand Faisal Hamad Mohamed, Yahya Maghoub, Tanweer Hussain, Ijaz Kamal, Zubair Anwer, Mubarak Ariyo Bidmos, Abdel-Naser Elzouki

**Affiliations:** 1 Division of General Internal Medicine, Weill Cornell affiliated-Hamad General Hospital, Hamad Medical Corporation, Doha, Qatar; 2 Weill Cornell College of Medicine, New York and Doha, Qatar; 3 College of Medicine (QU Health), Qatar University, Doha, Qatar; 4 Department of Diabetes and Endocrinology, Wirral University Teaching Hospital NHS Foundation Trust, Arrowe Park Road, Upton, Wirral, CH49 5PE Liverpool, United Kingdom; 5 Acute Medicine Unit, Derbyshire Hospitals Foundation Trust, Derby, England United Kingdom

**Keywords:** Avoidability; preventability; DILI; LAAT; elderly patients

## Abstract

**Background:**

Drug-induced liver injury (DILI) represents an increasing morbidity in the general population, but more so in the elderly cohort of patients. Despite this, the concept of its prevention through prospective analysis has largely remained unexamined. We evaluated the utility of recently validated adverse drug reactions (ADR) avoidability tool in a cohort of elderly patients with DILI.

**Methods:**

We examined 38 DILI-drug pairs from n=38 patients in a prospective cohort of patients presenting with adverse drug reactions to a Weill Cornell-affiliated tertiary hospital between February 2019 and January 2020. DILI outcomes were adjudicated by the updated Roussel Uclaf Causality Assessment Method (RUCAM). Two clinical pharmacologists and two general physicians utilized the Liverpool adverse drug reactions avoidability tool (LAAT) and the modified Hallas tools to rate the preventability of DILI-drug pairs. Inter-rater, exact agreement proportions, as well as intraclass correlation coefficients were generated and expressed as ordinal outcomes.

**Results:**

The cases examined for the determination of DILI avoidability had probability likelihood of “probable” or “highly probable” by the updated RUCAM scale. Examination of the 38 DILI-drug pairs (n= 38 patients) resulted in a total of 152 ordinal outcome decisions. We found about 32.3% (50/152) and 34.2% (52/152) of DILI-drug pairs were rated as “avoidable” (“probable” or “definite”) by the LAAT and the modified Hallas tools respectively. The overall median Krippendorf’s kappa with the LAAT was 0.61 (SE 0.12, CI 0.36, 0.85) and for modified Hallas tool was 0.53 (SE 0.18; CI 0.16, 0.89). The inter-rater correlation coefficient (ICC) for the LAAT and modified Hallas were 0.50 [0.32, 0.65] and 0.63 [0.48, 0.76] respectively. Exact pairwise agreement was present in 30/38 (IQR 29.5, 34.5), and 28/38 (IQR 27.5-35.5) of DILI-ADR pairs using the LAAT and modified Hallas tools respectively.

**Conclusion:**

We found a significant proportion of drug-induced liver injury adjudicated by the updated RUCAM scale in elderly hospitalized cohort of patients were avoidable with significant implication for therapeutic commissioning as well as cost effectiveness interventions in this cohort of patients.

## Background

There has been a demonstrable rise in the prevalence of drug-induced liver injury (DILI) in the general population, but more so within the elderly population cohort [1–4]. The elderly are particularly vulnerable to this morbidity due to a number of factors including polypharmacy that inevitably accompanies multi-morbidity with advancing age [5]. Added to this are the pharmacokinetic peculiarities associated frailty and the aging process including but not limited to vulnerabilities in drug absorption, distribution, and metabolism [5]. The exact prevalence of DILI in the elderly population remains unknown, but it is estimated from recent reports to range between 23-45% [6]. This uncertainty derives from the paucity of studies exploring the exact phenotype and burden of DILI in the older population cohort. Reducing the burden of DILI will be useful in the overall management of issues related to multi-morbidity-driven polypharmacy in this vulnerable population cohort. Amongst a range of recently suggested interventions is the determination of whether DILI cases in the elderly population were in fact “avoidable” *ab initio*.

Determining DILI drug classes that are potentially avoidable in the elderly will assist in rational therapeutic decision making, therapeutic commissioning, as well as the design of preventive measures aimed at reducing them [7]. In the general population, the modified Hallas [8] and more recently the validated Liverpool adverse drug reactions avoidability tool (LAAT) have increasingly been utilized in the determination of preventability of adverse drug reactions [7, 9]. Despite the rising burden of DILI-related morbidities in the elderly population, the concept of its prevention through formal systematic analyses have gone unexamined until now. In this study, we have for the first time attempted to evaluate the comparative potential utility of the modified Hallas and LAAT in the determination of DILI avoidability in the elderly population cohort. Determining the avoidability of DILI in the elderly population will be important in assisting geriatricians, policymakers, and clinical therapeutic commissioners in identifying drug classes accounting for significant morbidities that could be amenable to tailored interventions in order to reduce the burden.

## Methods

**Fig. 1:**
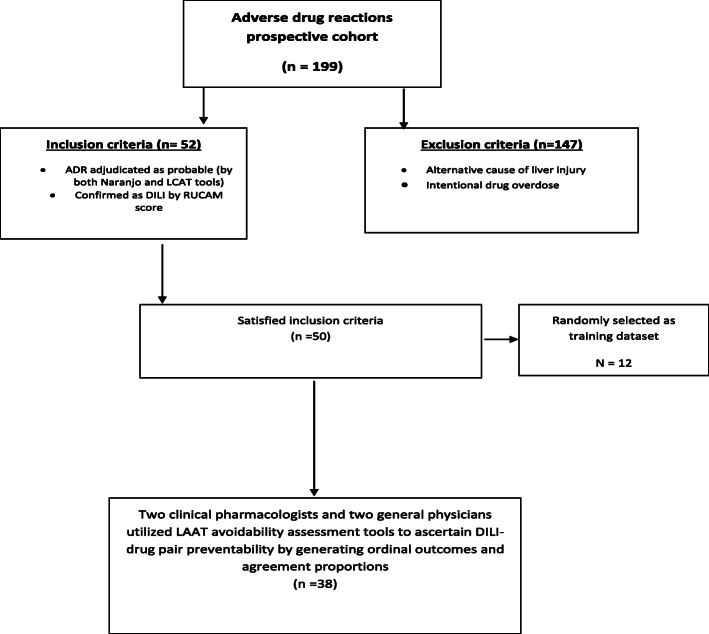
Flow chart of DILI-ADR case ascertainment

**Fig. 2:**
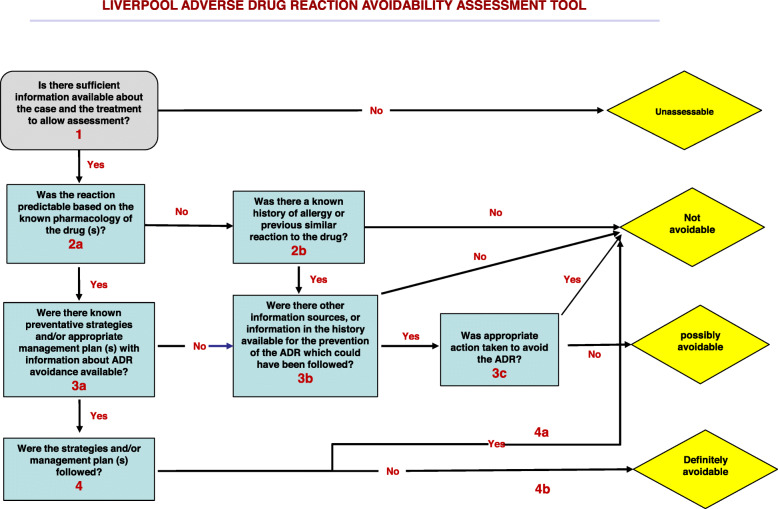
Schematic representation of the Liverpool adverse drug reactions avoidability tool showing the path of determination of avoidability (adapted from Bracken et al [9])

The study cohort comprised of all patients presenting to the Emergency Department (ED) or inpatient units of Weill Cornell Medicine-affiliated Hamad General Hospital (HMC) with suspected adverse drug reactions (ADR) as part of an ongoing prospective study cohort study (Fig. 1). For this sub-study, two reviewers considered patients >65 years from this cohort who were first adjudicated to have “probable” or “highly probable” DILI scores by the updated Roussel Uclaf Causality Assessment method (RUCAM) [10]. This is a universally acceptable, internationally validated structured tool that allocates specific scores to designated patient-drug interaction variables resulting in a quantitative grading of causality of DILI or herb induced liver injury (HILI) adjudication tool. The final score is interpreted thus: Drug-DILI pairs with scores of “0” or <0 indicate that the drug is “excluded” as a cause of DILI or HILI; 1 to 2 indicates that DILI/HILI is “unlikely”; 3 to 5 indicates “possible”; 6 to 8 “probable”; and >8, “highly probable”. We accepted and included DILI-drug pairs that are classed as “probable” or “highly probable” on the updated RUCAM scale. We subsequently utilized the updated scale tailored for the determination of hepatocellular injury for cases with R-ratio (described under case definitions below) of >5; and that specific for the adjudication of cholestatic or mixed injury for cases with R-ratio <2.5, or between 2.5-5. We then utilized the LAAT (Fig. 2), and modified Hallas tools to determine the potential avoidability of these DILI-drug pairs.

We abstracted the following demographic and clinical parameters of each patient (from an online patient data repository to a Microsoft excel database. These include ethnicity, sex, age, current medication, drug allergies, co-morbidities, index DILI-drug pair, and implicated drug. Other parameters include the dates of the following variables: ADR event, drug commenced, ADR drug stopped, and the onset of symptoms/signs. We also abstracted information on the resolution of ADR, the results of any investigations for differential diagnoses of the suspected DILI-drug pair, any documented record of DILI-ADR outcome, as well as if there was any record of any re-challenge. Amongst the range of alternative diagnoses (other than DILI) that we sought clarification include viral/autoimmune hepatitis, non-alcoholic fatty liver disease (NAFLD), liver and biliary ultrasound, as well as if patients have had a liver biopsy.

We excluded cases where there were alternative diagnoses of liver injury (other than the drug) and in cases of deliberate self-harm. We initially piloted the modified Hallas [8] and LAAT [9] tools on 12 randomly selected DILI-dug pairs. This was done for the dual purpose of training the prospective raters, as well as to improve the familiarity of the raters with these tools. We utilized two rating pairs (two clinical pharmacologists, and two general physicians) to rate the modified Hallas and the LAAT tools with the view to ascertain avoidability of DILI-drug phenotype pairs. In the unlikely event where raters require additional information for case ascertainment and causality determination, they were advised to access publicly available portals and databases of therapeutics information {such as Summary of Product Characteristics (SmPC), the European Medicines Agency (EMA); Food and Drug Administration (FDA); Medicines and Healthcare Products Regulatory Agency (MHRA)}. We reported DILI-drug outcomes measures as one of four-point ordinal scale i.e. ‘definitely avoidable’, ‘possibly avoidable’, ‘not avoidable’ and ‘unassessable’. We submitted and obtained ethical approval for this study from the independent review board of the Medical Research Centre (HMC) (Certificate number MRC-01-18-162).

### Statistical analyses

Continuous variables were expressed as means (±SD), or median (inter-quartile range) depending on distribution. Categorical variables were presented as numbers (percentages). We estimated the agreement proportions, Fleiss pairwise kappa (k), Krippendorf’s pairwise kappa (as appropriate), and intraclass correlation coefficients between the 38 DILI-ADR pairs.

DILI-ADR avoidability outcomes were classified as categorical variables, with their resulting pairwise interrater agreement proportions, Krippendorf’s kappa statistics with 95% confidence intervals (CI), and intraclass correlation coefficients (ICC). We estimated and compared the exact pairwise agreement and the disagreement between raters in order to determine agreement proportions across multiple raters. All statistical analyses were carried out with GraphPad Prism (version 8.00 for Windows, 2019 GraphPad Software, La Jolla California USA).

### Case definitions

- We define extreme agreement (EA) between a pair of raters where a particular DILI-ADR pair was scored to the same outcome [11]
- Where DILI-ADR pairs are scored to discordant outcomes by rating pairs; i.e. where one rater scores a DILI-ADR drug pair outcome as “unassessable”, with the other rating pair scoring it as any of the other possible ordinal outcomes (“not avoidable”, “possibly avoidable”, or “definitely avoidable”)
- We accept Kappa values of ≤ 0.20, 0.21–0.40, 0.41–0.60, 0.61–0.80, and 0.81–1 as representing a slight, fair, moderate, substantial, and almost perfect agreement, respectively [11]
- DILI was defined as alanine aminotransferase (ALT) or aspartate aminotransferase (AST) levels greater than 5 × the upper limit of normal (ULN) on two consecutive occasions (at least two weeks apart) and/or ALP levels greater than 2 × the ULN on two consecutive (at least two weeks apart) occasions [10]
- R-ratio: This index assists in the classification of liver injury into potentially hepatocellular, obstructive, and mixed categories. Itis calculated by dividing multiples of the upper limit of the normal ranges of alanine aminotransferase (ALT) by the alkaline phosphatase (ALP). Preferably, values used should be from blood samples that were drawn from the same day (or no more than 48 hours apart). Cases with values of greater than 5, less than 2.5, and between 2.5 to 5 are classified as hepatocellular, obstructive, and mixed Liver injury respectively. It is an initial first step in the estimation of RUCAM scores.

## Results

The case ascertainment flow chart as well as characteristics of the study population (*N* = 38) are shown in Figure 1 and Table 1 respectively. The mean age of the study population was 69 (7.63±) years. The study cohort comprised of predominantly male population, *N* = 21 (55.3%), with the principally implicated drugs been antimicrobial agents. The median R-score was 4, with an IQR [2, 4] suggestive of a “mixed liver injury” phenotype of this study population. Table 2 shows the disposition of various classes vis-à-vis DILI-drug pairs. The detailed updated RUCAM likelihood outcomes are presented in supplementary material (Table 3).

**Table 1:** Demographic characteristics of the study population (*N* = 38)

	All Medications
Age in years (SD)	69 (±7.63)
Male N (%)	21 (55.3)
Non-Arab nationality N (%)	15 (39.5)
Pattern of Liver injury	
Hepatocellular N (%)	6 (15.8)
Mixed N (%)	29 (76.3)
Cholestatic N (%)	3 (7.9)
Latency-time to onset of injury (days)	25.6 (±23.2)
Use of alcoholic beverages N (%)	3 (7.5)

**Table 2:** The distribution of DILI-ADR pairs by drug classes

N (%)	
**Antimicrobial agents**	**12 (31.5)**
**Anticonvulsants**	**7 (18.4)**
**Statins**	**7 (18.4)**
**Analgesics**	**5 (13.2)**
**Antihypertensives**	**7 (18.4)**
**Total**	**38 (100)**

**Table 3:** Updated RUCAM score proportion of cases in the study cohort (*N* = 40)

Likelihood	Frequency (N)	%
Highly Probable	15	37.5
Probable	21	52.5
Possible	1	2.5
Unlikely	0	0
Excluded	3	7.5

### DILI-ADR avoidability outcomes

Both the LAAT and modified Hallas tools resulted in a total of 152 DILI-ADR outcome decisions (across the two rating pairs) each. Of these, 32.3% (50/152) and 34.2% (52/152) were rated as “avoidable” (“probable” or “definite”). The proportion of “unassessable” outcomes reported with the modified Hallas tool across all raters (21.2%) was significantly higher (at P = >0.0001) than the LAAT tool (6.25%). This suggests the superiority of the LAAT tool over the modified Hallas tool as a preventability adjudication tool.

### Inter-rater agreement and reliability

**Fig. 3:**
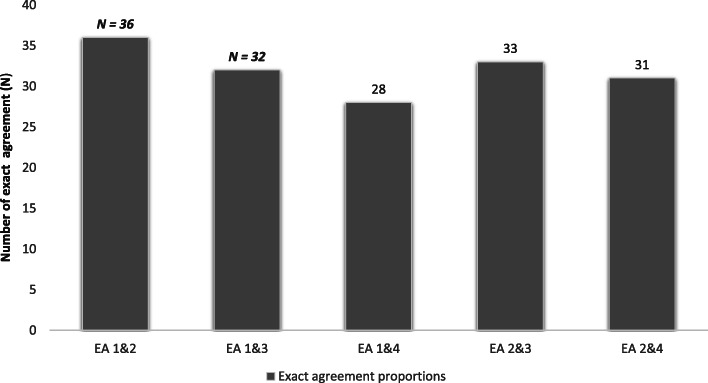
Proportion of exact agreement (EA) between rating pairs utilising the LAAT tool (Krippendorff’s alfa = 0.50 ICC = 0.73 [CI 0.55-0.85])

**Fig. 4:**
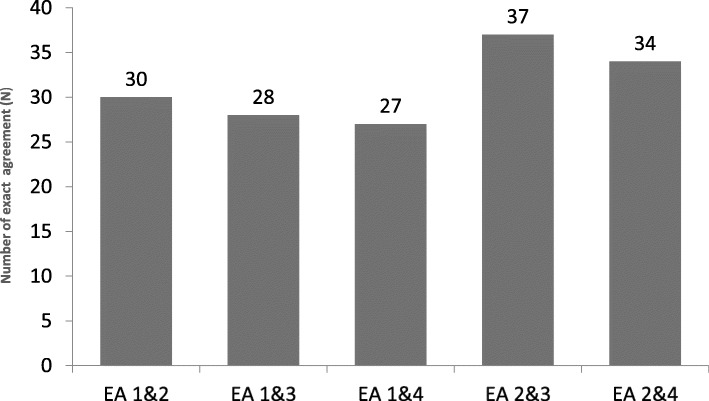
Exact agreement (EA) proportions between rating pairs utilizing the Hallas tool (Krippendorff’s alfa 0.40; ICC = 0.41 [CI = 0.22, 0.61])

The intraclass correlation coefficient (ICC) utilizing the LAAT tool was 0.50 (CI 0.32, 0.65), compared with the modified Hallas tool 0.63 (CI 0.48, 0.76). The overall median Krippendorf’s kappa with the LAAT was 0.61 (SE 0.12, CI 0.36, 0.85), and 0.53 (SE 0.18; CI 0.16, 0.89) with the modified Hallas tool. Exact pairwise agreement was present in 30/38 (IQR 29.5, 34.5), and 28/38 (IQR 27.5-35.5) DILI-ADR pairs using the LAAT and modified Hallas tools respectively. Exact agreement (EA) proportions amongst the clinical pharmacologists and general physicians were 85% (ICC 0.73, CI 0.55-0.85), and 77.5% (ICC 0.14, CI -0.15-0.42) respectively with the LAAT tool. Extreme disagreement (ED) occurred in 12.5% and 10% of DILI-ADR pairs that were rated with LAAT and modified Hallas tools respectively (Figs. 3 and 4).

## Discussion

This represents the first published attempt to our knowledge aimed primarily at exploring the preventability of drug-induced liver injury (DILI) in the elderly patient cohort. We observed that about 31-33% of all DILI-ADR pairs in this patient population were in fact avoidable (“probable” or “definite”). This is supported by moderate interrater reliability (IRR) of between 0.4 and 0.65. The interrater agreement and consistency among raters reported from our study is in agreement with findings from recently published reports that examined the utility of the novel LAAT tool in the general patient population [7, 9]. The result of this study demonstrates the potential for preventability of costly and mortality-prone drug-related liver injury in vulnerable elderly population especially those with multi-morbidity, although this needs to be confirmed in by prospective systematic studies.

Until recently, the exact role of aging process in the determination of the prevalence of DILI has always been a subject of intense epidemiological debate (10, 15). For example, despite the incorporation of an extra score for age (>55 years) in the RUCAM score [12], a single report failed to conclusively associate disproportionate burden of DILI with the elderly population [10]. However, recent data from population-based studies such as that reported by Björnsson et al. [13] have shown a clear correlation of DILI incidence with age. In this study (15) in which cases were based on RUCAM assessment, increasing age (>70 years) was associated with an estimated four-fold increased incidence of DILI. One of the reasons for this observation is that frailty and aging process are associated with increased susceptibility to intrinsic and idiosyncratic DILI through shifting of dose–response (DR) curve (to the left) and narrowing of the therapeutic window [14]. Additionally, multi-morbidity associated with the aging process confers additional polypharmacy burden to the already established inherent therapeutic challenges in the elderly population. Our data suggest that regardless of the avoidability adjudication tool utilized, almost one out of every three DILI cases in the elderly were potentially avoidable. This observation is strongly supportive of the updated RUCAM scale that incorporates age >55 years as one of the risk factors (with a score of +1) in the causality algorithm for both hepatocellular and cholestatic DILI [10]. It is very instructive from our study that despite our patient population cohort of >65 years, the updated RUCAM scale performed reasonably well, we suspect this may due incorporation of the extra score of 1 in the updated RUCAM algorithm.

In the current study, we found no significant difference in the proportion of exact agreement (EA) and exact disagreement (ED) between the two rating specialties (general physicians and clinical pharmacologists). Our choice of general physicians is deliberate as it reflects the population of physicians that interface daily with DILI-related morbidities, and often had to make clinical and therapeutic decisions without robust supporting risk stratification tools (such as the LAAT tool). Clinical pharmacologists were utilized to provide a comparative platform to assess whether in-depth therapeutic knowledge will impact on the usability of the tool. Our result suggests that this was not the case. The percentage of EA from our study (80%) is consistent with that reported by the original developers of the tool [9], and a recent study by Danjuma et al. [7]. In the study by Danjuma et al. [7], the percentage pairwise agreement utilizing the LAAT and modified Hallas tools were reported as 78.5% and 62.2% respectively.

The differences in our study population (elderly) compared to the patient population from which the LAAT tool was developed (pediatric oncology patients) appeared not to impact significantly on its performance. It has been argued that the relative preponderance of neutropenic patients in the study by Bracken et al. [9] may have “rail-roaded” a proportion of the ADR cases to be rated as “not avoidable” [7, 9]. We found no significant concerns regarding the disposition of “not avoidable” outcomes in our study. The rating clinicians in our study found the interface provided by the tool as satisfactory with adequate information that was consistent with what they encountered in the course of their daily practice. This observation is important taking into cognizance the potential utility of this tool as an additional layer of therapeutic risk stratification in the older category of patients.

We found no validated scheme for the training of raters on the use of the avoidability tools. Our random sampling and allocation of 12 DILI-drug cases for use by raters to familiarize themselves with the avoidability tools (over a week) enhanced their ability to subsequently score real DILI-ADR cases. The phenotype of DILI-ADR pairs in our report is representative of the common causes of DILI encountered on a daily basis in typical clinical wards or encounters. Up to a third of our DILI cases were attributable to antimicrobial agents with greater than three-quarters of this due to the antibiotic ceftriaxone. Previous reports from prospective registry data have estimated the attributable burden of antibiotics to the overall DILI-related morbidity between 12 and 18% [10]. This will particularly be useful especially in our current drive for antibiotic stewardship, and more work may be needed in this area to specifically investigate preventability of antibiotic related DILI in this population group of patients.

Reducing the burden of DILI in the elderly population is central to addressing the overall morbidity and sometimes unacceptable mortality attributable to therapeutics in this vulnerable population. The determination of whether these DILI-related phenotypes are in fact avoidable *ab initio* will be a force multiplier in this case. It will enable amongst others the fashioning of tailored made interventions targeting implicated drugs by both local, regional, and national therapeutic commissioning bodies.

### Strengths and Limitations

Our study represents the first attempt at exploring the concept of preventability of drug-induced liver injury in the elderly population. Its use of a recently validated ADR avoidability tool was seminal and complements physician/geriatrician’s clinical judgement in making holistic therapeutic decisions regarding this vulnerable patient group. The study is limited by its small sample size and therefore studies with adequate sample sizes are needed to corroborate our findings. Additionally, it was limited (as was the case with previous reports utilizing this tool) by the lack of standardization of training provided to the raters on how to use the tool. This will continue to generate concern until a reliable and dependable training scheme on the use of this tool is developed. Similarly, there remains lack of consensus to date on the exact cutoff for kappa scores that represent optimal IRR. However, our kappa score of >6 will represent moderate IRR and will be within the “ball-park” recommended by developers of the tool.

## Conclusion

We found a significant proportion of drug induced liver injury in elderly hospitalized cohort of patients were avoidable. This, if validated by further prospective studies will have significant implication for therapeutic commissioning, and cost effectiveness analyses in these cohorts of patients.

## Supplementary information

**Additional file 1: Table S1.** Individual DILI-drug pairs with updated RUCAM causality grading.

## Data Availability

The datasets used and/or analyzed during the current study are available from the corresponding author on reasonable request.

## Abbreviations

ADR: Adverse drug reactions
ALP: Alkaline phosphatase
ALT: Alanine aminotransferase
AST: Aspartate aminotransferase
DILI: Drug induced liver injury
EA: Exact agreement
ED: Emergency Department
FDA: Food and drug administration
EMA: European medicines agency
ICC: Intraclass correlation coefficients
IRR: Interrater reliability
LAAT: Liverpool adverse drug reactions avoidability tool
LCAT: Liverpool causality assessment tools
MHRA: Medicines and healthcare products regulatory agency
MRC: Medical research centre
NAFLD: Non-alcoholic fatty liver disease
RUCAM: Roussel Uclaf Causality Assessment Method
ULN: Upper limit of normal
SmPC: Summary of product characteristics
