# Supplementary material for: Avoidability of drug-induced liver injury (DILI) in an elderly hospital cohort with cases assessed for causality by the updated RUCAM score
Source: BMC Geriatr. 2020 Sep 14;20:346. doi: 10.1186/s12877-020-01732-3 (PMC7489200; doi:10.1186/s12877-020-01732-3)
Supplement: Supplementary file 1 — Additional file 1: Table S1. Individual DILI-drug pairs with updated RUCAM causality grading. [file 12877_2020_1732_MOESM1_ESM.docx]

**Supplementary material**

**Table S1:** Individual DILI-drug pairs with updated RUCAM causality grading

| Likelihood | | | | | | Comments |
| --- | --- | --- | --- | --- | --- | --- |
| DILI-Drug pairs | **Highly probable** | **Probable possible** | **Possible** | **Unlikely** | **Excluded** |  |
| Ceftriaxone | X |  |  |  |  |  |
| Ceftriaxone |  | x |  |  |  |  |
| Rosuvastatin | X |  |  |  |  |  |
| Co-amoxiclav |  | x |  |  |  |  |
| Piperacillin/tazobactam |  | x |  |  |  |  |
| Simvastatin | X |  |  |  |  |  |
| Levetiracetam |  | X |  |  |  |  |
| Moxifloxacin |  | X |  |  |  |  |
| Phenytoin | X |  |  |  |  |  |
| Paracetamol | X |  |  |  |  |  |
| Diclofenac |  | X |  |  |  |  |
| Lacosamide |  | X |  |  |  |  |
| Azithromycin |  | x |  |  |  |  |
| Rifaximin | X |  |  |  |  |  |
| Sodium valproate |  | x |  |  |  |  |
| Atorvastatin | X |  |  |  |  |  |
| Isoniazid |  | X |  |  |  |  |
| Rifampicin | X |  |  |  |  |  |
| Simvastatin | X |  |  |  |  |  |
| Phenytoin |  | X |  |  |  |  |
| Rifampicin | X |  |  |  |  |  |
| Levetiracetam |  | X |  |  |  |  |
| Aspirin |  | X |  |  |  |  |
| Ceftriaxone |  | X |  |  |  |  |
| Amlodipine |  |  |  |  | x |  |
| Phenobarbitone | X |  |  |  |  |  |
| Ceftriaxone |  | X |  |  |  |  |
| Felodipine |  |  |  |  | x |  |
| Hydralazine |  | X |  |  |  |  |
| Aspirin |  | x |  |  |  |  |
| Ramipril |  |  |  |  | x |  |
| Atorvastatin | X |  |  |  |  |  |
| Nifedipine |  |  | x |  |  |  |
| Sodium valproate | X |  |  |  |  |  |
| Simvastatin | X |  |  |  |  |  |
| Hydralazine |  | X |  |  |  |  |
| Paracetamol | X |  |  |  |  |  |
| Pravastatin |  | X |  |  |  |  |
| Hydralazine |  | X |  |  |  |  |
| Atorvastatin |  | X |  |  |  |  |
